# Band gap closure, incommensurability and molecular dissociation of dense chlorine

**DOI:** 10.1038/s41467-019-09108-x

**Published:** 2019-03-08

**Authors:** Philip Dalladay-Simpson, Jack Binns, Miriam Peña-Alvarez, Mary-Ellen Donnelly, Eran Greenberg, Vitali Prakapenka, Xiao-Jia Chen, Eugene Gregoryanz, Ross T. Howie

**Affiliations:** 1 Center for High Pressure Science Technology Advanced Research, 1690 Cailun Road, Shanghai, 201203 China; 2 Centre for Science at Extreme Conditions, School of Physics and Astronomy, University of Edinburgh, Edinburgh, EH9 3FD UK; 3 Center for Advanced Radiation Sources, University of Chicago, Chicago, IL 60637 USA

**Keywords:** Structure of solids and liquids, Inorganic chemistry, Phase transitions and critical phenomena

## Abstract

Diatomic elemental solids are highly compressible due to the weak interactions between molecules. However, as the density increases the intra- and intermolecular distances become comparable, leading to a range of phenomena, such as structural transformation, molecular dissociation, amorphization, and metallisation. Here we report, following the crystallization of chlorine at 1.15(30) GPa into an ordered orthorhombic structure (*oC*8), the existence of a mixed-molecular structure (*mC*8, 130(10)–241(10) GPa) and the concomitant observation of a continuous band gap closure, indicative of a transformation into a metallic molecular form around 200(10) GPa. The onset of dissociation of chlorine is identified by the observation of the incommensurate structure (i-*oF*4) above 200(10) GPa, before finally adopting a monatomic form (*oI*2) above 256(10) GPa.

## Abstract (web summary)

Molecular systems are predicted to transform into atomic solids and be metallic at high pressure; this was observed for the diatomic elements iodine and bromine. Here the authors access the higher pressures needed to observe the dissociation in chlorine, through an incommensurate phase, and provide evidence for metallization.

## Introduction

Under sufficient compression, all molecular systems are expected to collapse into close-packed metals. This process was most notably predicted to occur for hydrogen, leading to a condensed metallic phase exhibiting exotic properties such as the simultaneous dissipationless transfer of matter and electricity^1–3^. On compression, hydrogen is predicted to go through a sequence from molecular insulator, to molecular metal and finally to an atomic metallic state. The experimental realisation of atomic metallic hydrogen has remained elusive despite intense research efforts lasting over 30 years^4–7,8,9–15^. However, this high-pressure phase transition sequence has been realised in the heavy halogens, iodine (I_2_) and bromine (Br_2_), providing an insight into the nature of homonuclear diatomic molecular systems at extreme conditions^16–19^. Intriguingly, the halogens have been found to exhibit remarkable phase progression en route to dissociation, with molecular metallisation, incommensurate structures, and superconductivity observed.

The behaviour of the lighter halogens, fluorine (F_2_) and chlorine (Cl_2_), forms a link between the phenomena observed for bromine and iodine and that of hydrogen. Although several experimental works on the lighter halogens exist up to moderate pressures (~50 GPa)^20,21^, they fall significantly short of the pressures required to observe either metallisation or the onset of dissociation. Previous empirical extrapolations, based on the heavier halogens, suggest that pressures approaching several million atmospheres (~250 GPa) would be required to dissociate chlorine^22^. The technological challenges associated with small sample sizes at these conditions (<10 μm^3^) combined with the complications of working with extremely hazardous chemicals, has ultimately left the nature of dissociation in the lighter halogens a subject of theoretical speculation^23,24^.

Chlorine is widely used in the industrial production of plastics and is naturally abundant in chloride salts with the Cl^−^ anion contributing ~2% of the oceans’ mass. The most familiar of these chloride salts, rocksalt (NaCl), exhibits highly unusual properties under moderate pressures^25^. In the presence of excess Na or Cl, new stable compounds with modified stoichiometries are reported, which seemingly violate any traditional chemical understanding. These works have further gone on to stimulate research into halogen chemistry at high pressures to investigate unusual chemical compositions of halide materials^26,27^. Critically, the identification of these materials has relied on the corroboration between theoretical calculation and experimental observation, with the former heavily reliant on accurate data of the pure systems which is lacking for chlorine and fluorine.

**Fig. 1:**
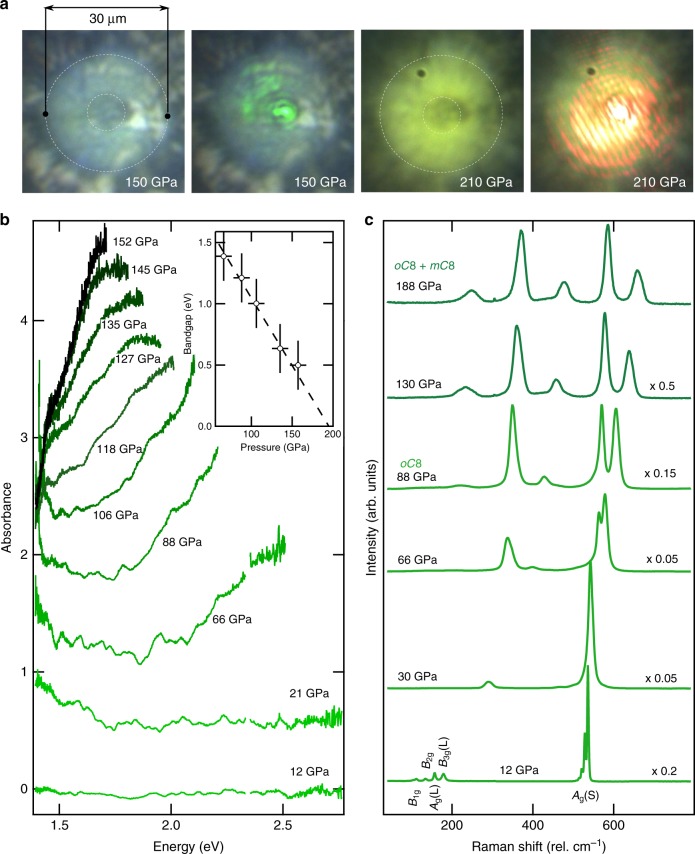
Optical absorption and Raman measurements up to 200 GPa. **a** Micrographs of chlorine demonstrating strong reflectance of 532 nm and 660 nm light at 150 and 210 GPa, respectively. **b** Representative absorption spectra for different pressures up to 135 GPa. Linear extrapolations of the absorption provide a tentative band gap value^52^. Inset—band gap measurements, deduced from the host figure, as a function of pressure, the extrapolation (solid black line) indicates band gap closure will occur in phases *oC*8/ *mC*8 at ~200 GPa. Error bars are ±0.2 eV and ±10 GPa. **c** Raman spectra below 200 GPa illustrating a significant redistribution of Raman intensities between internal (*A*_g_(S) and *B*_3g_(S)) and external modes (*A*_g_(L) and *B*_3g_(L). The coexistance of phases *oC*8 and *mC*8, denoted by the dark green colour, is marked by the asymetric profile of the modes coinciding with the emergence of the lowest frequency *B*_1g_(L)-2 mode, see Fig. 2

**Fig. 2:**
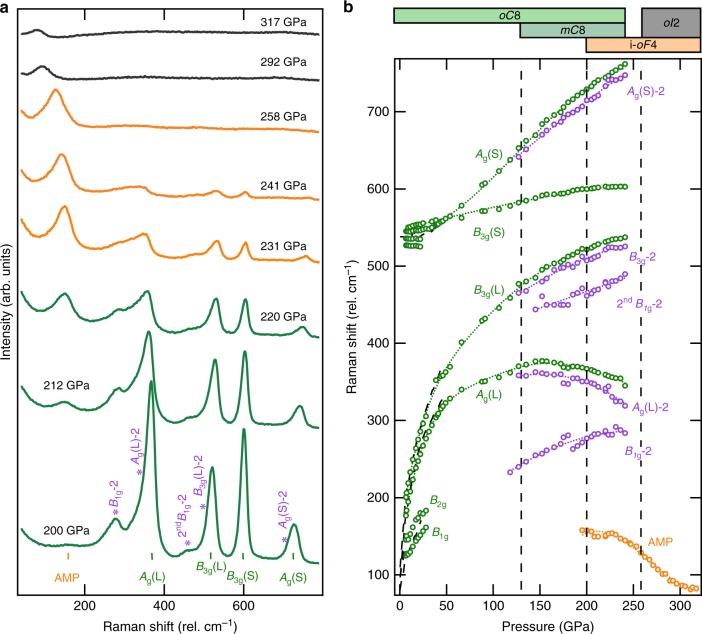
Raman measurements above 200 GPa. **a** Raman spectra collected of solid chlorine up to 317 GPa. Excitations corresponding to *oC*8 (green markers: *A*_g_(L), *B*_3g_(L), *B*_3g_(S), *A*_g_(S)), *mC*8 (purple markers: *B*_1g_(L)-2, *A*_g_(L)-2, *B*_3g_(L)-2, *A*_g_(S)-2) and the amplitude mode (orange markers: AMP) of the *i-oF*4 phase are denoted below the lowest pressure spectra. **b** Frequencies of excitations present in solid chlorine as a function of pressure, black dashed curves are from ref. ^20^. The vertical dashed lines denote phase transitions, with the corresponding 1-D phase diagram provided atop of the figure describing the evolution and coexistance found in chlorine’s phase behaviour. The abundance of each phase in coexistance can be qualitatively approximated from the intensities of their excitations, seen here in panel **a** and quantified in Supplementary Figure 2b

**Fig. 3:**
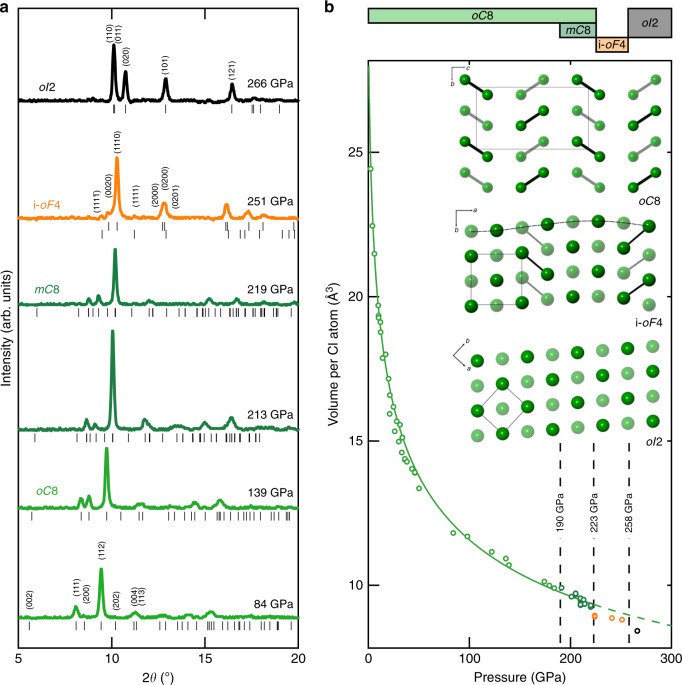
X-ray diffraction measurements. **a** High-pressure X-ray diffraction patterns (*λ* = 0.3344 Å) of chlorine with increasing pressure. At 251 GPa, chlorine adopts an incommensurate structure based on a face-centred orthorhombic parent phase whose reflections are marked with the upper set of tick marks. Satellite reflections are shown with the lower set of tick marks. At 266 GPa chlorine has transformed to an atomic phase crystallising in a body-centred orthorhombic unit cell; **b** volume per Cl atom as a function of pressure. A third Birch-Murnaghan equation of state^53^ was used to describe the pressure evolution (*V*_0_ = 29.1222 Å, *K*_0_ = 8.3(11) GPa, *K*’ = 5.6(4)), denoted by the solid green curve, to the molecular *oC*8 and *mC*8 phases. The 1-D phase diagram atop of the figure provides the phase behaviour of chlorine as observed with X-ray diffraction. Inset—crystal structures in the *b*–*c* plane of *oC*8, *i-oF*4 and *oI*2 of chlorine, differences in the opacity represent atoms belonging to different layers in the structures. Top panel—*oC*8 (*Cmce*), where distinct differences in intermolecular and intramolecular distances are observed. Middle Panel—*i-oF*4, a modulated structure as a precursor to complete molecular dissociation. Bottom panel—*oI*2 (*Immm*), the simple close-packed structure of atomic chlorine

In this work, we subject chlorine to ultra-high pressures, in excess of several million atmospheres (>300 GPa), using a suite of complimentary techniques for characterisation: transmission measurements (Fig. 1a), Raman spectroscopy (Figs. 1b and 2) and X-ray diffraction (Fig. 3). We identify a continuous band gap closure, indicative of the onset of molecular metalicity in the halogens, and observe the continuous dissociation of Cl_2_ molecules through an incommensurate phase before adopting a purely atomic form at 258(10) GPa. This study presents insights into the dissociation of elemental molecular systems at extreme conditions.

## Results and discussion

### Crystallisation and the *oC*8 phase of chlorine

After loading, chlorine was found to crystallise at 1.15(30) GPa, shown in Figs. 1b and 3, in agreement with recent work^28^. The transition was confirmed by X-ray diffraction with peaks that were indexed to an orthorhombic space group *Cmce* [*a* = 5.8655(7), *b* = 4.1937(2) and *c* = 7.9455(4) Å] with four chlorine molecules per unit cell. Referred to in this study by its Pearson notation, *oC*8, this low-pressure phase is found to be isostructural to phase I of both iodine and bromine^22^. The transition is marked by the appearance of sharp lattice modes in the Raman signature, opposed to the broad distribution of rotational modes characteristic of unhindered chlorine molecules in the fluid state (see Supplementary Figure 1). As the lowest frequency modes, *B*_1g_ and *B*_2g_, and isotopic differences in the *A*_g_(s) mode become unresolvable at pressures greater than 20 GPa, seen in Fig. 1b, only four modes are discernible in the Raman spectra for this structure at high pressures: two stretching modes (*A*_g_(S) and *B*_3g_(S)) accompanied by two librational modes (*A*_g_(L) and *B*_3g_(L))^20,24^. The pressure dependency of these modes have been tracked up to the dissociation of chlorine, 258(10) GPa, where the Raman spectra exhibit no molecular excitations. The *oC*8 phase displays remarkable pressure stability and to our knowledge extends over the largest pressure range (223(10) GPa) for any molecular system, for comparison phase I of hydrogen exists for a pressure interval of only 180 GPa^29^. Over this extensive pressure domain the atomic volume of chlorine is found to reduce by more than 75% (−19.8 Å^3^), see Fig. 3b. The large volume collapse is a manifestation of the significant reduction in the intermolecular distances as opposed to any profound intramolecular changes, reflected by the relatively weak pressure dependence of the internal stretching modes (*A*_g_(S) and (*B*_2g_(S)) in comparison with the external modes (*A*_g_(L) and *B*_3g_(L), seen in Fig. 2b.

### The *mC*8 phase of chlorine

At higher pressures, *oC*8 was found to coexist with the emergence of a previously unreported phase, *mC*8, and was identified by subtle changes in X-ray diffraction patterns. As found for I_2_^30^, a *C*-centered monoclinic structure (space group *C*2/*m*) [*a* = 2.847(1), *b* = 4.190(2), *c* = 7.118(2) Å and *β* = 113.83(3)° at 205 GPa], improved residuals and explained the misfits of several reflections. Interestingly, the structure comprises two different types of intramolecular covalent bonds, a characteristic seen in other diatomic systems and is believed to be the mechanism behind the rich vibrational spectra reported in dense hydrogen above 200 GPa^29^. The onset of the *mC*8 phase is more readily identifiable through Raman spectroscopic measurements, seen above 130 GPa as the development of peak asymmetry and the emergence of new peaks associated with the *B*_1g_(L)-2, *A*_g_(L)-2, *B*_3g_(L)-2 and *A*_g_(S)-2 excitations, identified in Fig. 2. The *B*_3g_(S)-2 mode however, could not be resolved due to insignificant differences with the *B*_3g_(L) mode inherent to the *oC*8 phase, analogous to theoretical simulations of iodine and bromine^30,31^. As with *oC*8, *mC*8 is remarkably stable, with both found to be in coexistence up to the appearance of the atomic phase at 258 GPa, again similar behaviour is observed in the analogous phases of iodine and bromine^30,31^. As expected the *Cmce* (*oC*8) and the *C*2/*m* (*mC*8) structures are found to be energetically competitive, reporting free energies and enthalpy differences of <3 and <5 meV/atom, respectively in the case of bromine^31^.

### Evidence for molecular metallisation

One of the most fascinating phenomena in solid-state science is the pressure-induced transition from an insulating to conductive state, particularly the transitions concerning covalently bonded systems. Here, the first observation of the onset of this phenomena in chlorine is seen by the significant changes of the relative of intensities amongst the internal/stretching *A*_g_(S) mode with the other inherent vibrations. As seen in Figs. 1c and 2a, the *A*_g_(S) mode is found to proceed through a maximum in intensity, first evolving to be ~20 times larger than the lattice modes, before becoming comparable with the other excitations at 88 GPa, seen in Fig. 1b. As the intensity of Raman excitations are strongly correlated to the charge densities involved in each of the respective modes^32^, these relative changes are therefore indicative of the first signs of an electronic redistribution inherent to a continuous metallisation. As a more direct probe, transmission measurements report the continuous closure of the band gap in chlorine, with the sample visually appearing dark at 50 GPa, with a measured band gap of ~0.5 eV at 150 GPa and extrapolated band gap closure at 200(10) GPa (see Fig. 1a). Therefore, the closure of the band gap in chlorine is found to occur at a pressure approximately an order of magnitude higher than that of iodine (~20 GPa)^17^ a direct consequence of the increased bond strength and therefore the electron localisation in the respective molecules. In accordance with the other members of the halogen group (summarised in Supplementary Figure 4), the band gap closure is not associated with any structural transition or dissociation, and is found to be purely a modification of its electronic structure.

### The incommensurate *i-oF*4 phase of chlorine

Evidence of incommensurate structures in elemental systems are scarce with the halogens providing the only diatomic molecular examples. In this study, the first evidence of the appearance of an incommensurate phase in chlorine, *i-oF*4 (‘*i*’ indicates that the structure is incommensurate), is identified spectroscopically around 200 GPa, with the appearance of a excitation, seen in Fig. 2. Here, the new mode is denoted as AMP, for the amplitude mode—an excitation inherent to incommensurate structures and is always Raman active^33^, seen clearly in Fig. 2 and Supplementary Figure 2. The direct structural identification of *i-oF*4 was found at higher pressures, 223 GPa, and can be seen in Fig. 3. The strongest peaks could be indexed to a face-centered orthorhombic cell [*a* = 3.034(1), *b* = 2.9893(7), *c* = 3.947(3) Å], indicating an abrupt drop of 4.4 % (0.4 Å^3^) in average atomic volume. These main reflections were accompanied by additional weak satellite peaks, (1,1,11̄,) and the (1,1,1̄,1) in Fig. 3, due to the structural modulation. Through an analogy with iodine^16^, the incommensurate nature of *i-oF*4 is accounted for by small shifts in atomic position creating a transverse wave along the *a*-axis with a modulation vector **k** = (0.230(5),0,0) at 241(10) GPa. The refined modulation vector was found to be in broad agreement with iodine phase V^16^, with both structures denoted as *Fmm*2(*α*00)0*s*0 in terms of the superspace-group notation. The apparent disagreement between X-ray and Raman diagnostics of the onset of this phase, a discrepancy of ~30 GPa, can be addressed by coexistence as is expected for a first-order phase transition. As can be seen in Fig. 2a, b and Supplementary Figure 2, there is large coexistence of *i-oF*4 with the precursory phases *oC*8/ *mC*8 and the successive purely atomic phase, *oI*2 (discussed later). For a first approximation an intensity analysis of Raman excitations has been conducted to find the relative abundance of each phase, summarised by Supplementary Figure 2b, revealing an exponential extinction of the modes related to phases *oC*8/ *mC*8, concomitantly with a significant growth of the AMP mode. Conversely, at higher pressure, the AMP mode has a marked decline in intensity after the identification of the *i-oF*4 phase by X-ray diffraction. Therefore, *i-oF*4 is only found to be the dominant phase for 223 to 258 GPa, which is only ~25% of its overall pressure stability, seen in Figs. 2, 3 and Supplementary Figure 3.

An abundance of elemental incommensurate structures have been reported at moderate pressures in groups 15–17, close to the divide in the periodic table between metallic and non-metallic systems, including the heavier halogen iodine^34–38^. To our knowledge, the chlorine *i-oF*4 phase, is the highest pressure observation of an incommensurate structure, the previously being Na-*i-tI*19 existing up to 180 GPa^39,40^. Despite having such contrasting physical properties at ambient conditions—Na being a nearly-free electron metal and Cl_2_ a molecular insulator, both materials show the critical role that incommensurate phases play during transitions associated with profound alterations in electronic structures at very high densities. In Na, the *i-tI*19 phase exists on the cusp of the metal-to-insulator transition^40^ and here the *i-oF*4 phase at the insulator-to-metal transition discussed previously.

### Dissociation and the *oI*2 phase of chlorine

At 241 GPa, all excitations present in the Raman spectra inherent to a molecular characteristic of chlorine are lost, see Fig. 2a,b and Supplementary Figure 2b, indicative that the former molecular phases *oC*8 and *mC*8 no longer coexist and only the modulated *i-oF*4 remains. Further, by 266(10) GPa just four strong reflections are apparent in the X-ray diffraction pattern, these peaks can be indexed to a body-centred orthorhombic cell, space group *Immm* [*a* = 3.7720(11), *b* = 2.0287(3), *c* = 2.2003(4) Å] accompanied by another small drop in atomic volume, ~4.5 % (0.8 Å^3^). In this structure, chlorine atoms lie on each lattice point with no clear molecular bonding, signalling the entrance to its first purely atomic phase, *oI*2. Rietveld refinement of this structure showed good agreement with the data, the final agreement factor at 266(10) GPa is *wR*_obs_ = 13.99% (Supplementary Figure 3). The transition from a molecular to atomic form is apparent from the nearest-neighbour interatomic distances which converge from 1.989(18), 2.223(16), and 2.525(15) Å at 138(10) GPa to 2.0337(6), 2.2038(7), and 2.4097(6) Å (at 266(10) GPa), becoming comparable to the intramolecular bond length of 1.994(2) Å at ambient pressure^41^.

The structural transition, identified by X-ray diffraction, is found to happen simultaneously with a significant drop in intensity of the only remaining excitation in the Raman spectra, the AMP-mode, at 258 GPa. Indicative that despite remnants of the *i-oF*4 phase, the bulk of the chlorine atoms adopt the *oI*2 phase. Additionally, there is strong evidence that this phase may become superconducting at low-temperatures, as both isostructural iodine and bromine have been reported to be superconducting at ~1.5 K^19^. Theoretical calculations suggest a significantly enhanced critical temperature in chlorine for this structure, ~4 K^24^, however reporting the emergence of *oI*2 at a much lower pressure^24^. As chlorine readily reacts with common electrode materials, definitive determination of the critical temperature and its underlying mechanisms would require non-contact measurements such as monitoring its nuclear magnetic response^42,43^.

It is interesting, that despite large regions of coexistence, exemplified by only a third of chlorine’s isotherm exhibiting solitary phase behaviour, we find there is no coexistence between the purely molecular phases, (*oC*8 and *mC*8), with the atomic phase, *oI*2. As observed previously in iodine, the appearance of the modulated structure, *i-oF*4, here again is found to serve as nature’s intermediary between molecular and atomic systems. The structural modulation gives rise to a continuous distribution of interatomic distances, seen in the inset to Fig. 3b, resulting in a state where there is no clear distinction between molecular and atomic units. This is in marked contrast to some proposed models presented for hydrogen which consist of mixed atomic-molecular phases^29^. Critically, significant differences between theoretical expectation and experimental observation are found, with dissociation previously proposed in chlorine at 157 GPa^24^, only ~60% of what is experimentally observed here. Therefore, this study further highlights the importance of experimental work to refine our chemical understanding of materials and it will consequently impact predictions for lighter halogen chemistry and dissociation, and perhaps that of other elemental diatomic molecules, most notably hydrogen.

## Methods

### Pressure generation and sample preparation

Diamond Anvil Cell (DAC) devices were used to generate the pressures, with diamond geometries varying from 250–30 μm culets to reach pressures of ~50 GPa to in excess of 300 GPa, respectively. The sample chambers were formed by a laser-milled cavity in a Re-foil, which is then sealed by the opposing anvils. The initial cavity dimensions are dependent on the diamond geometry, typically the Re-foil was indented to an initial thickness a tenth and the cavity laser-trepanned approximately a third of the culet diameter. Typically we find when these parameters were exceeded, sample confinement at higher pressures became unfavourable.

Prior to loading, chlorine was generated as a gas via an oxidation reaction between concentrated hydrochloric acid and manganese dioxide (Eq. 1). The effervesced gas was then cleansed of impurities such as hydrogen chloride and water, by channelling it first through a water bath and a drying agent powder, calcium chloride (CaCl_2_), respectively. The refined high-purity Cl_2_ gas was then passed over liquid nitrogen cooled diamond anvils, where it condensed in its solid form. Finally, the diamonds are brought together to confine the sample and recovered to ambient temperatures. The entirety of the procedure was conducted in a protective dry-nitrogen atmosphere. To ensure sample purity a highly sensitive long-exposure Raman probe was used to detect contamination and/or reactions with the gasket material.

1 $$4{\mathrm{HCl}}(\mathrm{l}) + {\mathrm{MnO}}_2(\mathrm{s}) \to {\mathrm{MnCl}}_2(\mathrm{s}) + 2{\mathrm{H}}_2{\mathrm{O}}(\mathrm{l}) + {\mathrm{Cl}}_2(\mathrm{g})$$

Noble metal pressure markers were not used in the experiments, due to the exceptional reactivity of Cl_2_ readily forming binary compounds. Ruby however, was found to be stable and no reaction was observed, therefore up to pressures of 50 GPa, pressure was determined by the calibrated shift of its R_1_ fluorescence line^44^. In the multi-megabar regime, where there is limited space for a pressure marker and the fluorescence response of the R1 ruby line become undetectable, the stressed diamond *T*_2*g*_ phonon^45^ is used, as is common practice for these pressures^29^. An example of the highest pressure stressed diamond *T*_2*g*_ profile is provided by Supplementary Figure 5.

As mentioned, Cl_2_ has a remarkable reactivity and therefore reactivity with the sample environment has to be considered. It has already been pointed out that there is potential for photochemistry with the gasket material. However, if not exposed to intense broadband laser light, Cl_2_ appears to show limited reactivity with its sample environment, i.e. the gasket and diamond anvils. In an effort to understand the sample-gasket chemistry, low-pressure high-temperature experiments were conducted to promote a reaction, see Supplementary Figure 1. A new vibrational mode corresponding to a Re-Cl stretch was found, indicating that a binary compound had been formed. These excitations however, had no correspondence with the excitations observed in Cl_2_ along it’s room temperature isotherm, ruling out the possibility of contamination. Further, depending on the state of the anvils after the conclusion of the experiments the culet surfaces were inspected under microscope and no etching, indicative of a chlorine-diamond reaction, was observed.

### Raman spectroscopy

Raman measurements were taken with a custom-built highly focussed Raman system, using both 532 and 660 nm laser sources. At pressures above 200 GPa solely a laser of 660 nm was used to by-pass the pressure-induced fluorescence emitted by the diamond anvils. Measurements were imaged on a CCD, with typical sample exposures of 10 s at 25 mW of incident laser power. The intensity vs wavenumber dataset was then background subtracted and accurate peak parameters were determined using the Fityk^46^ software package.

### X-ray diffraction

Powder X-ray diffraction (XRD) data were collected at the BL10XU (Spring-8, Japan)^47^ and GSECARS 13-IDD (Advanced Photon Source, USA)^48^ beamlines with the energies in the range of 30–42 keV. The imaged diffraction was integrated using the DIOPTAS software^49^ to yield two-dimensional intensity vs 2*θ* datasets. Patterns were indexed with *GSAS-II*^50^. The Le Bail and Rietveld refinements were carried out in Jana2006^51^. Finally, equation of state parameters were determined using the EoSFit7^52^ software package.

### Transmission measurements

Transmission measurements were taken by a broadband white-light LED lamp focussed through the back anvil and collected, collimated by a Mitutoyo objective being imaged by a CCD. Supercontiuum measurements were attempted, however after laser incidence instantaneous reaction with the gasket occurred forming a Re-Cl compound. The reaction resulting in visually changed sample as well as modified Raman signature.

## Supplementary information

Supplementary Information

Peer Review File

## Data Availability

The authors declare that the main data supporting the findings of this study are contained within the paper and its associated Supplementary Information. All other relevant data are available from the corresponding author upon reasonable request.
