# Author Correction: Band gap closure, incommensurability and molecular dissociation of dense chlorine

**DOI:** 10.1038/s41467-019-10187-z

**Published:** 2019-05-03

**Authors:** Philip Dalladay-Simpson, Jack Binns, Miriam Peña-Alvarez, Mary-Ellen Donnelly, Eran Greenberg, Vitali Prakapenka, Xiao-Jia Chen, Eugene Gregoryanz, Ross T. Howie

**Affiliations:** 1 Center for High Pressure Science Technology Advanced Research, 1690 Cailun Road, Shanghai, 201203 China; 2 Centre for Science at Extreme Conditions, School of Physics and Astronomy, University of Edinburgh, Edinburgh, EH9 3FD UK; 3 Center for Advanced Radiation Sources, University of Chicago, Chicago, IL 60637 USA

**Keywords:** Structure of solids and liquids, Inorganic chemistry, Phase transitions and critical phenomena

Correction to: *Nature Communications* 10.1038/s41467-019-09108-x, published online 08 March 2019.

The original version of this Article omitted references to previous experimental reports on solid hydrogen that are relevant for a full understanding of the context of the previous work. The added references are:

47. Akahama, Y. et al. Evidence from x-ray diffraction of orientational ordering in phase III of solid hydrogen at pressures up to 183 GPa. *Phys. Rev. B* **82**, 060101 (2010).

48. Zha, C.-S., Liu, Z. & Hemley, R. J. Synchrotron infrared measurements of dense hydrogen to 360 GPa. *Phys. Rev. Lett*. **108**, 146402 (2012).

49. Dias, R. & Silvera, I. Observation of the Wigner-Huntington transition to metallic hydrogen. *Science* **355**, 715–718 (2017).

50. Eremets, M. I. & Drozdov, A. P. Comments on the claimed observation of the Wigner-Huntington transition to metallic hydrogen. Preprint at http://arxiv.org/abs/1702.05125 (2017).

51. Loubeyre, P., Occelli, F. & Dumas, P. Comment on: “Observation of the Wigner-Huntington transition to metallic hydrogen”. Preprint at http://arxiv.org/abs/1702.07192 (2017).

52. Goncharov, A. F. & Struzhkin, V. V. Comment on “Observation of the Wigner-Huntington transition to metallic hydrogen”. *Science* **357**, eaam9736 (2017).

53. Liu, X.-D., Dalladay-Simpson, P., Howie, R. T., Li, B. & Gregoryanz, E. Comment on “Observation of the Wigner-Huntington transition to metallic hydrogen”. *Science* **357**, eaan2671 (2017).

Citations to these reference, plus reference 21, have been added to the fourth sentence of the Introduction: ‘The experimental realisation of atomic metallic hydrogen has remained elusive despite intense research efforts lasting over 30 years^4–7,21,47–53^.’ This has been corrected in the PDF and HTML versions of the Article.

