# Supplementary material for: Band gap closure, incommensurability and molecular dissociation of dense chlorine
Source: Nat Commun. 2019 Mar 8;10:1134. doi: 10.1038/s41467-019-09108-x (PMC6408506; doi:10.1038/s41467-019-09108-x)
Supplement: Supplementary file 1 — Supplementary Information [file 41467_2019_9108_MOESM1_ESM.pdf]

Band Gap Closure, Incommensurability and Dissociation of Dense Chlorine

Supplementary Information

Philip Dalladay-Simpson[1], Jack Binns[1], Miriam Peña-Alvarez[2], Mary-Ellen Donnelly[1], Eran Greenberg[3], Vitali Prakapenka[3], Xiao-Jia Chen[1], Eugene Gregoryanz[1,2,4] and Ross T. Howie[1],

[1]Center for High Pressure Science Technology Advanced Research, Shanghai, People's Republic of China

[2] Centre for Science at Extreme Conditions and School of Physics and Astronomy, University of Edinburgh, Edinburgh, UK

[3] Center for Advanced Radiation Sources, University of Chicago, IL, 60637, USA

[4] Key Laboratory of Materials Physics, Institute of Solid State Physics, Chinese Academy of Sciences, People's Republic of China

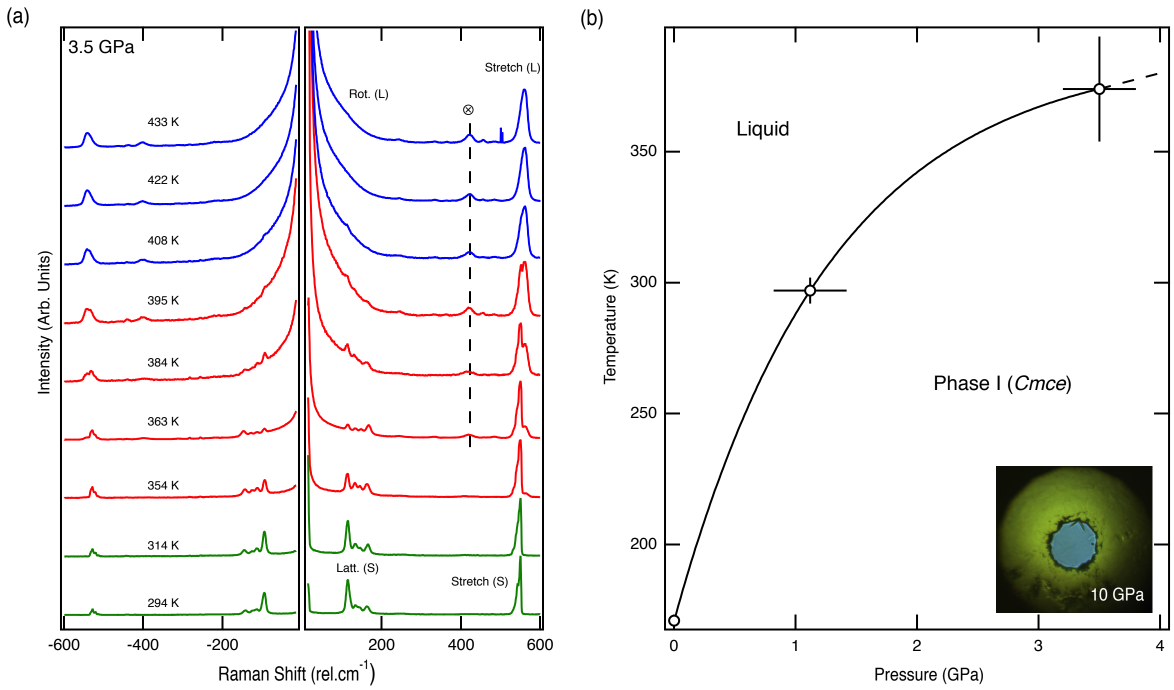

Supplementary Figure 1 (a) Representative Raman of a heating run on Cl_2 at 3.5 GPa showing both Stokes and Anti-Stokes contributions. The waterfall displays the evolution of the melting, initially in the solid *Cmce* phase (green), followed by an observation of the coexistance with the liquid phase (red) and finally reaching a pure liquid state (blue). The dashed line, marked by the circle, indidicates the region where an Re-Cl stretch is observed. (b) Melting curve of Cl_2 up to 4 GPa and 375 K. Error bars are ±0.3 GPa, room temperature crystallisation is ±5 K and within the heating experiment ± 20 K. Inset - micrograph of chlorine in both transmitted and reflected light at 10 GPa on a 200μm culet.

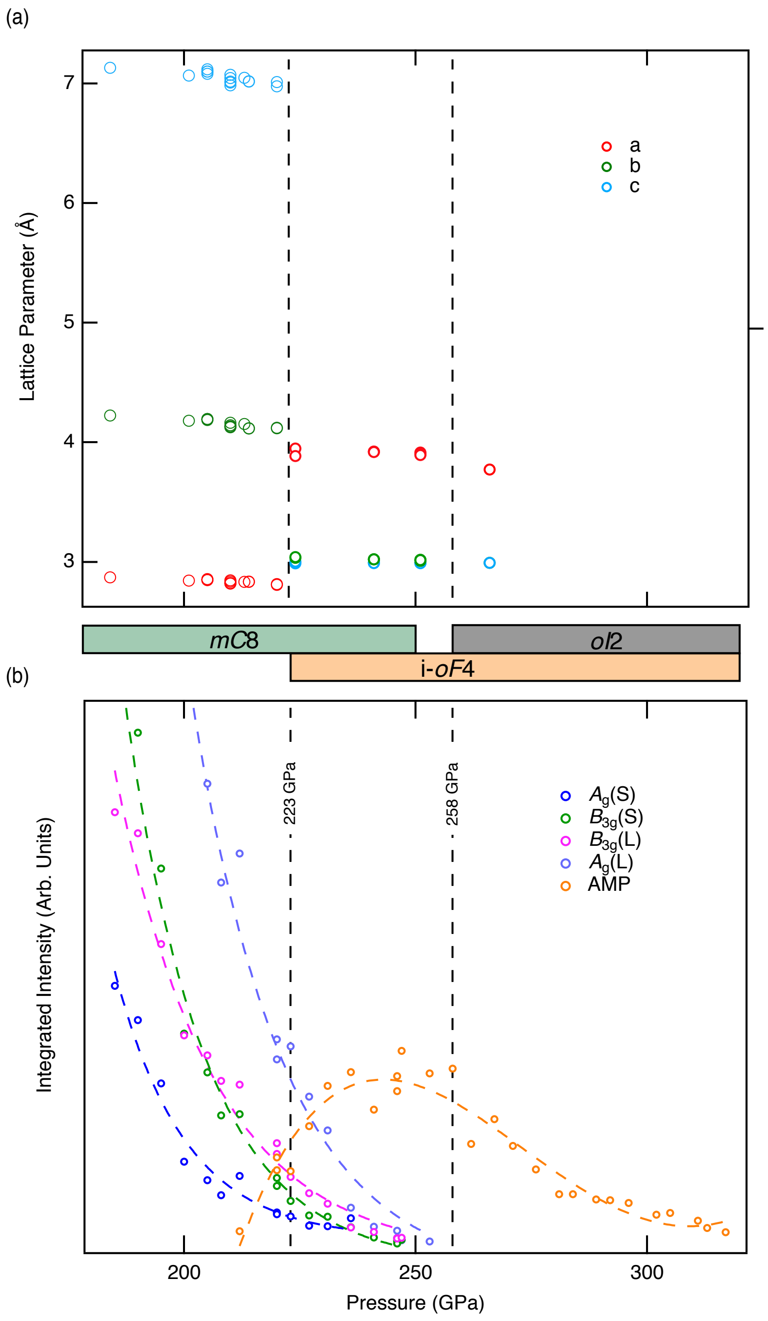

Supplementary Figure 2 (a) Lattice parameters for dense chlorine, phase transitions as seen by X-rays are denoted by dashed lines and correspond well to the integrated intensities as seen in Raman.(b) Integrated intensities of the excitations found in chlorine at multi-Mbar pressures. The intensities of modes present in phase I, are well described by exponential pressure dependence with simulateous extinction reported in the stability region of the incommensurate phase, denoted as phase V. The amplitude of the AMP mode, characteristic of the modulation of the incommensurate structure, phase V, is observed to process through a maximum, before rapidily decreasing in intensity on molecular dissociation to phase II.

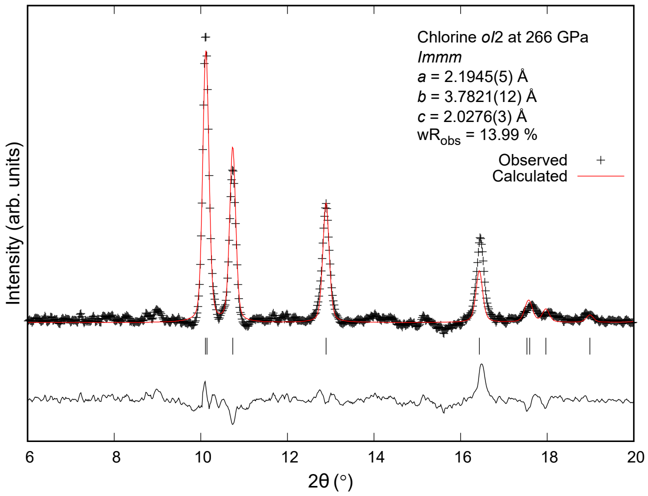

Supplementary Figure 3 Full structure Rietveld refinement of $oI2$ phase. Black crosses, red solid lines and black lines represent experimental, modelled and difference spectra. The ticks below profile indicates the predicted peak positions. Agreement factor for this data is $wR_{obs} = 13.99\%$, $\lambda = 0.3344$ Å

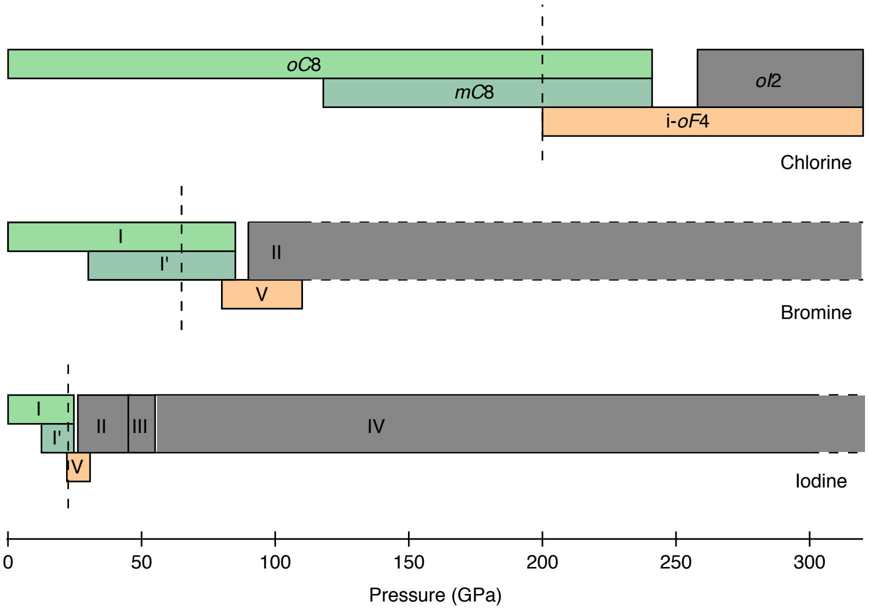

Supplementary Figure 4 Review of phase transitions in the halogens along the room temperature isotherm, anologous phases are denoted in the same colours and from refereces [1–3]. The molecular phases, $oC8$ and $mC8$ (coloured in green), are isotructural to the molecular phases I and I' of iodine and bromine. The inconmensurate structures of the haolgens are denoted in orange, whilst the atomic phases are in grey. The horizontal dashed lines in each of the phase diagrams define the entrance molecular-metallic state.

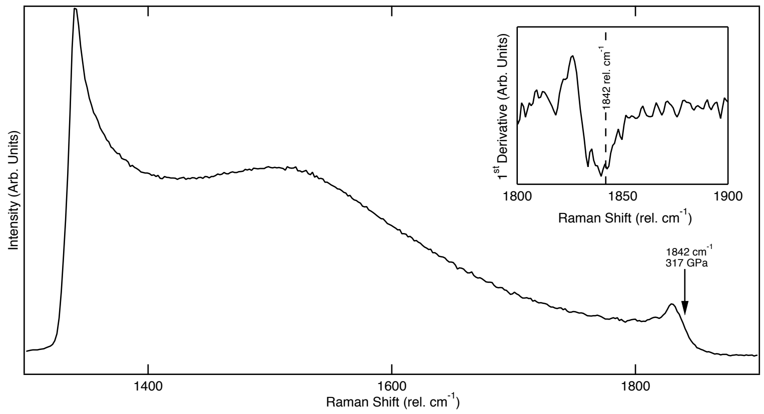

Supplementary Figure 5 Representative Raman spectrum of the stressed diamond T_{2g} phonon, with an edge with a frequency of 1842 cm^{-1} corresponding to a pressure of 317(10)GPa[4]. Inset - a first derivative of the spectrum in the host figure between 1800-1900 cm^{-1}.

[1] Fujii, Y. *et al.* Evidence for molecular dissociation in bromine near 80 GPa. *Physical Review Letters* **63**, 536–539 (1989).

[2] Kume, T., Hiraoka, T., Ohya, Y., Sasaki, S. & Shimizu, H. High pressure raman study of bromine and iodine: Soft phonon in the incommensurate phase. *Physical Review Letters* **94**, 1–4 (2005).

[3] Kenichi, T., Kyoko, S., Hiroshi, F. & Mitsuko, O. Modulated structure of solid iodine during its molecular dissociation under high pressure. *Nature* **423**, 971–974 (2003).

[4] Akahama, Y. & Kawamura, H. Pressure calibration of diamond anvil Raman gauge to 410 GPa. *Journal of Physics: Conference Series* **215** (2010).
