# Supplementary material for: Band gap closure, incommensurability and molecular dissociation of dense chlorine
Source: Nat Commun. 2019 Mar 8;10:1134. doi: 10.1038/s41467-019-09108-x (PMC6408506; doi:10.1038/s41467-019-09108-x)
Supplement: Supplementary file 2 — Peer Review File [file 41467_2019_9108_MOESM2_ESM.docx]

**Editorial Note**:

This manuscript has been previously reviewed at another journal that is not operating a transparent peer review scheme. The manuscript was considered suitable for publication without further review at Nature Communications.
